# Exploring readiness for implementing goal-oriented care in primary care using normalization process theory

**DOI:** 10.1017/S1463423622000767

**Published:** 2023-02-08

**Authors:** Ine Huybrechts, Dagje Boeykens, Agnes Grudniewicz, Carolyn Steele Gray, An De Sutter, Peter Pype, Dominique Van de Velde, Pauline Boeckxstaens, Sibyl Anthierens

**Affiliations:** 1 Department of Family Medicine and Population Health, University of Antwerp, Antwerp, Belgium; 2 Department of Family Medicine and Chronic Care, Free University of Brussels, Brussels, Belgium; 3 Department of Rehabilitation Sciences, Occupational Therapy, Faculty of Medicine and Health Sciences, Ghent University, Ghent, Belgium; 4 Department of Public Health and Primary Care, Faculty of Medicine and Health Sciences, Ghent University, Ghent, Belgium; 5 Telfer School of Management, University of Ottawa, Ottawa, Ontario, Canada; 6 Bridgepoint Collaboratory for Research and Innovation, Lunenfeld-Tanenbaum Research Institute, Sinai Health System, Toronto, Ontario, Canada; 7 Institute of Health Policy, Management & Evaluation, Dalla Lana School of Public Health, University of Toronto, Toronto, Ontario, Canada; 8 End-of-Life Care Research Group, Faculty of Medicine and Health Sciences, VUB and Ghent University, Ghent, Belgium; 9 Department of Occupational Therapy, Artevelde University of Applied Sciences, Ghent, Belgium

**Keywords:** goal-oriented care, implementation, normalization process theory, primary care

## Abstract

**Aim:**

To use normalization process theory (NPT) to build a strategy for the implementation of goal-oriented care (GOC) in primary care in Flanders, Belgium.

**Background:**

GOC is a possible approach to more coordinated and integrated care and tailors care to patients’ personal life goals. The concept has gained interest among policy makers and researchers, but the main drivers for successful implementation are the primary healthcare professionals (PHCPs) who need to see added value of GOC in order to embed it into their daily practice. NPT, developed to understand the processes of implementing new ways of organizing care, offers a useful lens to understand adoption of GOC in primary care practice.

**Method:**

PHCPs (*n* = 131) who participated in a 2-hour community meeting on GOC were asked to complete the Normalization MeAsure Development survey. This 23-item survey is based on NPT and describes participants’ views about how an intervention would impact their work, their expectations about it, and whether it could become a routine part of their work.

**Findings:**

The NPT constructs coherence (sense-making work) and cognitive participation (relational work) showed positive tendency toward implementation of GOC. The participants had an initial understanding on GOC and there was much interest in supporting and start working with this approach. The other constructs collective action (operational work) and reflexive monitoring (appraisal work) will need further efforts to trigger implementation. A common ground is needed to integrate GOC as a common practice which can be achieved by intensive interprofessional collaboration.

## Introduction

Primary healthcare professionals (PHCPs) around the world often care for patients with numerous health and social needs. The increasingly complex combinations of diseases and social problems make it challenging to provide optimal care (Loeb *et al*., 2016; Starfield, 2011). Patients with multiple problems require care across diverse organizations and healthcare professionals leading to a risk of fragmented care (Boult and Wieland, 2010). Additionally, caring for patients with multiple problems can be burdensome and may not be centered around the patients’ personal needs. Consequently, the care provided to these patients can be at odds with what is actually needed: an integrated and patient-centered approach (Boult and Wieland, 2010; Kodner and Spreeuwenberg, 2002; Mold *et al*., 1991; Reuben and Tinetti, 2012). One of the possible strategies to more integrated, patient-centered care for patients is goal-oriented care (GOC), which tailors care to the patient’s personal life goals instead of to disease and problem-oriented targets (Mold *et al*., 1991; Reuben and Tinetti, 2012; Boeckxstaens *et al*., 2016; Schellinger *et al*., 2018). If professionals explicitly focus on the patient’s personal life goals, care could potentially be better aligned with what is most important to patients and lead to better integration of the care provided across the system (Mold, 2017; Gray *et al*., 2020).

While GOC is gaining interest in research and policy as a potential catalyst for integrated care (Gray *et al*., 2020; Reuben and Tinetti, 2012; World Health Organization, 2018; Charette *et al*., 2015; Mold, 2017), it remains unclear how PHCPs in the field feel about GOC and whether they would be willing and able to implement and embed – or normalize – GOC in their daily practice. Gaining insights in the perceptions of PHCPs about GOC is important to work toward successful implementation of this approach in the long term.

Normalization process theory (NPT) has been specifically developed to help understand and explain the processes of implementation and normalization of complex interventions, such as GOC (May and Finch, 2009; May, 2013; May *et al*., 2018; McEvoy *et al*., 2014; Murray *et al*., 2010; De Brún *et al*., 2016). NPT suggests that four constructs or mechanisms play a central role in embedding new processes of care: (1) coherence (whether the intervention makes sense to PHCPs), (2) cognitive participation (how PHCPs engage with it), (3) collective action (how they enact it), and (4) reflexive monitoring (how they appraise the ways that a new set of practices affect them and others around them) (NPT Core Constructs (May and Finch, 2009)). Studies that use NPT can identify which elements need the most effort and resources for the intervention to be successful from the development phase to the implementation phase. For example, NPT has been previously used as a theory to better understand implementation efforts for the implementation of person-centered care (Alharbi *et al*., 2014), for a complex intervention targeted at compassionate care (Bridges *et al*., 2017), and for the implementation of integrated care (Ling *et al*., 2012).

NPT states that practices become routinely embedded – or normalized – as the result of people working, individually and collectively, to enact them. NPT emphasizes the contribution and roles of individuals and groups in the implementation of new ways of organizing care. As the theory focuses on action rather than more intentions or attitudes (May, 2015), it can provide insight into how GOC is interpreted individually and collectively by PHCPs and how this is reflected in their routine practices.

This study aims to explore the potential for the implementation of GOC in primary care in Flanders, Belgium through an NPT lens. NPT allows to look into the mechanisms of change that are at stake when planning a local initiative to support the implementation of GOC in primary care. This study will capture how these limited implementation efforts will affect the four different constructs of NPT. What mechanisms are already triggered for GOC to be embedded in routine practices and what mechanisms still need further incitement when developing a sustainable implementation plan for GOC?

## Methods

### Context

In Flanders, Belgium, primary care is characterized by many self-employed PHCPs (such as family physicians, nurses, and physiotherapists), as well as attached PHCPs working in different organizations (community health centers, group practices), who are all organized and financed at different levels (local, regional, federal). As part of primary care reform efforts, in 2010, the Flemish government introduced local multidisciplinary networks to support PHCPs in implementing chronic disease management programs. As of December 2018, the local multidisciplinary network and clinician-researchers at Ghent University worked together to organize two-hour interprofessional meetings about GOC. These meetings (all similar) contained two parts: (1) an introduction on the concept of GOC relating to the disease-oriented paradigm and (2) a workshop using cases and role plays to complement the theoretical knowledge with practice-based examples and tools. It should be emphasized that these meetings were not considered as an intensive training on GOC, but they did offer a first impression on the topic which could have triggered participants to apply GOC in their practice. Attendance at these meetings was completely voluntary.

### Sample

For this study, purposive sampling was used; PHCPs working in Ghent were recruited at the end of the interprofessional community meeting on GOC. These PHCPs had limited experience or knowledge about GOC but showed interest in GOC as an innovative approach. Given this context and the intent of the survey described in the next section, these PHCPs were well-suited to reflect on the items of the Normalization MeAsure Development (NoMAD) survey.

### Data collection

The survey was introduced in three local community meetings in 2019. Immediately after the PHCPs attended one of the meetings, they were asked to complete the NoMAD survey on paper before they left the meeting (Finch *et al*., 2015). The NoMAD survey has been developed to assess the perspective of PHCPs who need to embed a new practice into their daily work through an NPT lens. The instrument is used to describe participants’ views about how a new practice would impact their work, their expectations about whether it could become a routine part of their work, and how it would influence collaboration within and outside of their organization (Finch *et al*., 2015).

Firstly, demographic data were collected, including the participants’ profession, gender, and age. Place of employment was not asked to assure anonymity. Secondly, three general statements on GOC were assessed and 22 items of the NoMAD were completed. These 22 items reflected the four NPT constructs: coherence (4 items), cognitive participation (4 items), collective action (9 items), and reflexive monitoring (5 items) (see Table 1). Two questions were added on top of the original NoMAD survey (collective action) to make it more applicable for the context of this study. These items were assessed by a 5-point Likert scale. Participants indicated an item as ‘not applicable’ when it was not relevant to their role, at this stage in their career, or for the intervention.

**Table 1.** Normalization process theory constructs

Core construct	Definition	Components
Coherence	Sense-making work done individually and collectively during operationalization of a set of practices	• Differentiation • Communal specification • Individual specification • Internalization
Cognitive participation	Relational work done to build and sustain a community of practice around a new practice	• Initiation • Enrollment • Legitimation • Activation
Collective action	Operational work done to enact new set of practices	• Interactional workability • Relational integration • Skill set workability • Contextual integration
Reflexive monitoring	Appraisal work done to assess and understand the effects of new practices on themselves and others around them	• Systematization • Communal appraisal • Individual appraisal • Reconfiguration

Finch T, Girling M, May C, Mair F, Murray E, Treweek S, *et al*. NoMAD: implementation measure based on Normalization Process Theory (Measurement instrument). Retrieved from http://wwwnormalizationprocessorg. 2015.

A validated Dutch version of the NoMAD survey was used (Vis *et al*., 2019). The survey has been established as highly reliable (20 items, α = 0.89), with a construct validity for coherence (4 items, α = 0.71), collective action (7 items, α = 0.78), cognitive participation (4 items, α = 0,81), and reflexive monitoring (5 items, α = 0.65) (Finch *et al*., 2018).

### Data analysis

The answers on the three general NoMAD items scored with the Visual Analogue Scale scale were converted to the 5-point Likert scale (categories 0 and 1 were integrated, as well as categories 2 and 3, 4 to 6, 7 and 8, and 9 and 10). Applicable for the 22 questions of the NoMAD, positive answers (agree or strongly agree) were compared to negative answers (disagree or strongly disagree). This approach eventually contributed to a better understanding of the results and is consistent with how the tool is meant to be scored (Finch *et al*., 2015).

The results are presented using frequencies. Based on these frequencies, data were interpreted allowing the broadest insights of the participants on the items of the NoMAD survey. Scores ranging from 75% and above were considered high (or scores below 25%, for those items that were asked negatively). Scores ranging from 55% to 75% were considered to represent a mildly positive result. Scores below 55% were considered to have a low result, meaning that these items represent a shortcoming or barrier for implementation. When more than half of the items within an NPT construct showed positive results, we argue that this construct is already ‘triggered’ which means that this will be beneficial toward GOC becoming a routinized and embedded practice. When several items within a construct showed more mixed or negative results, this means that the construct should still be triggered by further implementation efforts in order to normalize GOC as an approach. No further statistical analyses were conducted.

### Ethics

Approval of the ethical committee of Ghent University was obtained (approval number 2018/1489) and written informed consent of all participants was given before the start of the survey.

## Results

### Demographics

In total, 131 PHCPs participated in one of the three community meetings that were selected for this study as they felt in the timeline. After the meeting, 104 completed the survey (response rate = 79%). Table 2 gives an overview of the participants’ demographics.

### Initial experience and views on GOC

**Fig. 1:**
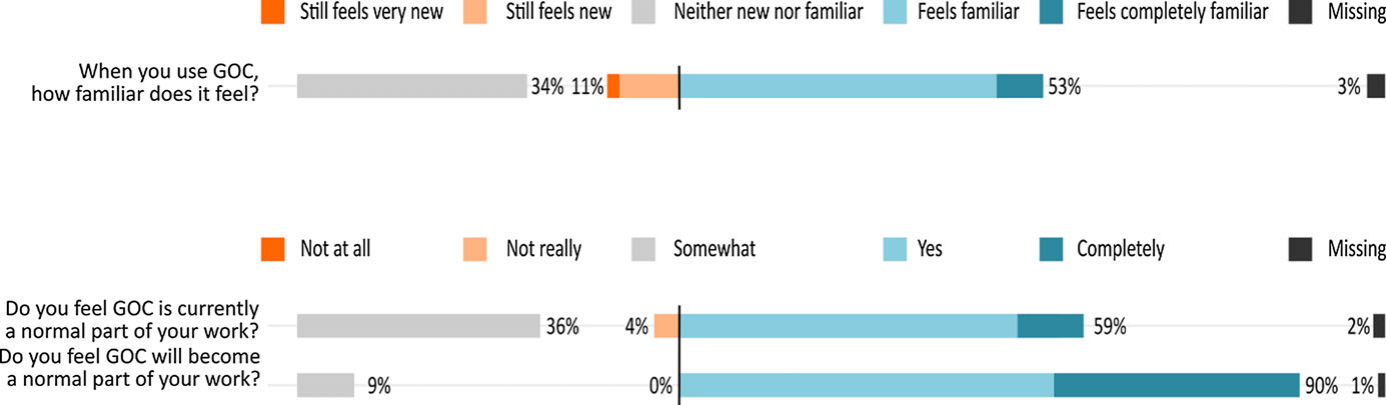
General NoMAD statements on goal-oriented care

The questions and results for how PHCPs experience and evaluate GOC are presented in Figure 1. Although the participants had limited knowledge on GOC as a concept, they often seem to recognize this way of working as an intrinsic part of their work: 53% of the participants reported feeling comfortable and familiar in using GOC and 59% felt that GOC is currently a normal part of their work. A majority of participants (90%) were optimistic that GOC will become a normal part of their work.

**Table 2.** **Demographics of participants (** ***n*** **= 104)**

Characteristics	***n*** (% of valid responses*)
Profession (*n* = 104)	
Nurse	18 (17.3)
Physiotherapist	18 (17.3)
General practitioner	15 (14.4)
Social worker	15 (14.4)
Pharmacist	9 (8.7)
Dietician	9 (8.7)
Certified nursing assistants	8 (7.7)
Other**	12 (11.5)
Gender (*n* = 104)	
Female	85 (81.7)
Male	19 (18.3)
Age group (*n* = 101, 3 missing)	
≤ 35	43 (42.6)
36–50	35 (34.7)
≥ 51	23 (22.7)

*% of responses excluding missing.

**2 occupational therapists, 2 midwives, 2 psychologists, 2 community center coordinators, 2 policy staff members, 1 community police officer, 1 expert by experience in poverty.

**Fig. 2:**
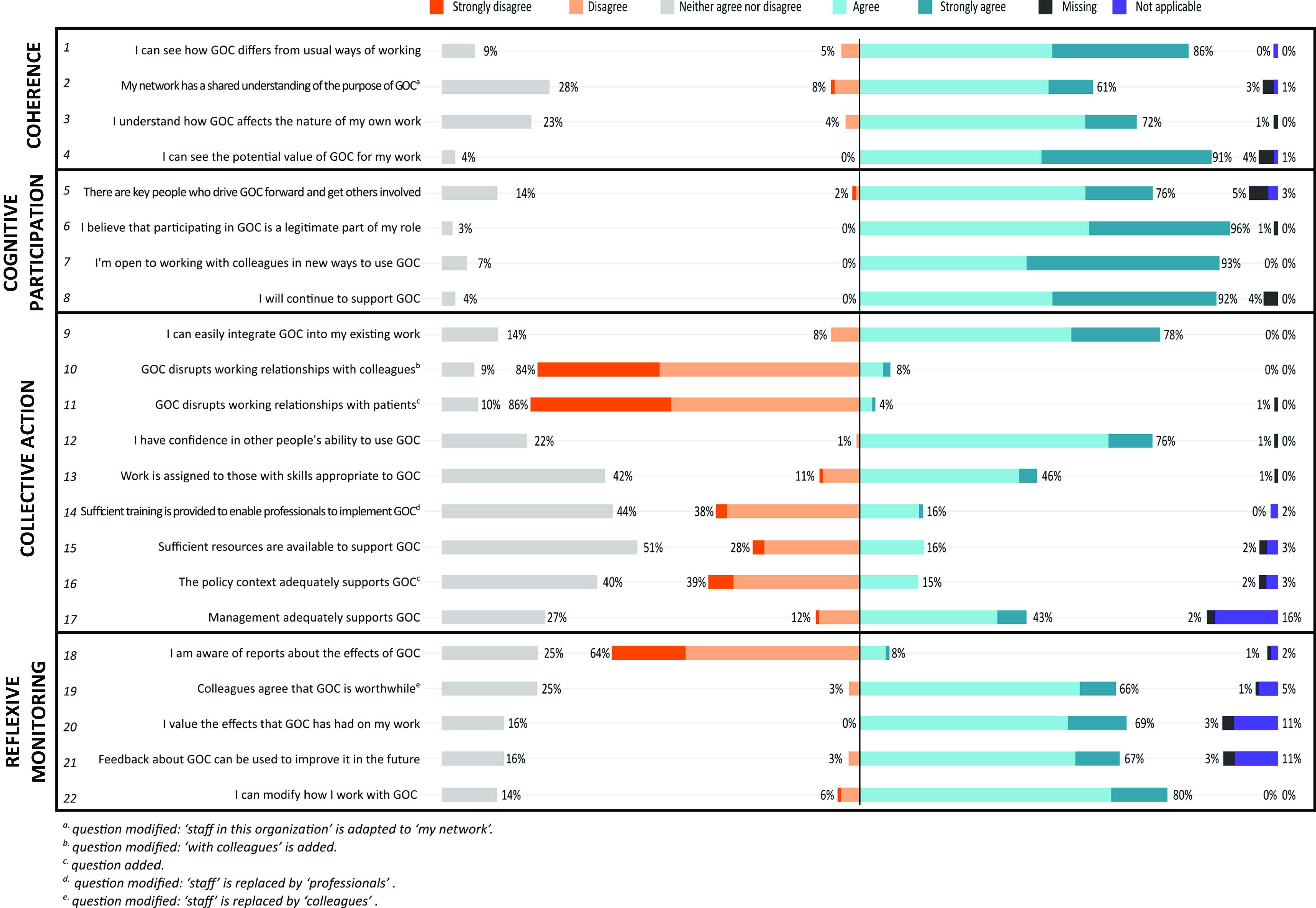
NoMAD statements on goal-oriented care

In what follows, the results of the four main constructs of the NPT (coherence, cognitive participation, collective action, and reflexive monitoring) are linked to the participants’ GOC experiences and views (May and Finch, 2009), as depicted in Figure 2.

### Coherence

Coherence is ‘*the sense-making work that people do individually and collectively when they are faced with the problem of operationalizing some set of practices* (Finch *et al*., 2015)’. It tells whether PHCPs adequately understand the nature of the intervention and how it fits into their current practices. Figure 1 illustrates that most participants felt they have a good idea about what GOC encompasses and how it can become an intrinsic part of their work. Most of them could clearly *differentiate* between a GOC approach and a traditional approach, with 86% of the participants reported that they recognize how GOC differs from usual ways of working. The large majority *internalized* the aims of GOC very well: 91% saw the potential value of GOC for their work. *Individual specification* (sense-making on the individual level (Finch *et al*., 2015)) was slightly lower: 72% of participants understood how GOC affects the nature of their own work. It appears that *communal specification* (collective sense-making (Finch *et al*., 2015)) is somewhat lower: 61% of participants believed that their network has a shared understanding of the purpose of GOC.

### Cognitive participation

Cognitive participation is about ‘*the relational work that people do to build and sustain a community of practice around a new technology or complex intervention* (Finch *et al*., 2015)’. This sample of participants showed a high engagement for adopting a GOC approach. Almost all PHCPs (96%) believed that participating in GOC is a *legitimate* part of their role. In terms of further *enrollment* or implementation of GOC, as well as the effects of GOC on team interactions, 93% reported to be open to working with colleagues in new ways to use GOC. Ninety-two percent of participants were willing to continue to support GOC, which is beneficial for further *activation* of GOC as an innovative practice. Somewhat more uncertainty is noticeable when it comes to the presence of key people who drive GOC forward and get others involved: 76% believed such key people are present in their working environment. According to the NPT methodology, key people are essential for *initiation* of such an intervention.

### Collective action

Collective action is ‘*the operational work that people do to enact a set of practices, whether these represent a new technology or complex healthcare intervention* (Finch *et al*., 2015)’. Seventy-eight percent of the participants reported they can easily integrate GOC into their existing work, meaning that *interactional workability* is rather high. When it comes to *relational integration*, participants did not believe GOC disrupts working relationships with colleagues (84%) or patients (86%). Furthermore, 76% of the participants had confidence in other people’s ability to use GOC. *Skill set workability,* allocating the work to those who are most fit for the given task (Finch *et al*., 2015), is more questionable. Only 46% of participants believed that work is assigned to those with skills appropriate to GOC. Moreover, only 16% of the participants reported that there is sufficient training provided to enable PHCPs to implement GOC. Around half of the participants (42% and 44%) neither agreed nor disagreed with these statements.

There was most uncertainty on the *contextual integration*, which is about resources and the presence or absence of a supporting policy and/or management context. Only 16% of participants was convinced that sufficient resources are available to support GOC. Fifty-one percent of participants neither agreed nor disagreed. Only 15% of participants believed that the policy context adequately supports GOC and 43% believed that management adequately supports GOC.

### Reflexive monitoring

Reflexive monitoring is ‘*the appraisal work that people do to assess and understand the ways that a new set of practices affect them and others around them (Finch et al., 2015)*’. This is an important measure for *systemization,* the work of collecting information to be able to assess the effectiveness and usefulness of GOC as an innovation. Only 8% was aware of reports on the effects of GOC (Finch *et al*., 2015). However, current evidence on the effectiveness of GOC is limited, which makes this measure rather irrelevant at this point*. Individual appraisal* gives a more positive image, with 69% of participants valuing the effect that GOC has had on their work. This is fairly similar to *communal appraisal,* with 66% of participants estimating that colleagues agree that GOC is worthwhile. According to how participants reported on the item representing *reconfiguration*, there seems to be openness when it comes to feedback about a GOC approach: 67% of participants believed that feedback about how GOC will be incorporated into daily practice can be used to improve it in the future. Moreover, 80% indicated that they will be able to modify how to work with GOC based on such feedback.

## Discussion

This study aimed to describe the potential for implementation of GOC in primary care in Flanders, Belgium using the NoMAD survey based on the NPT. The results of this study demonstrate that the multidisciplinary PHCPs in our sample showed understanding of and openness to implementing a GOC approach. First, coherence was rather high, as participants indicated that they have an initial understanding of GOC and recognize its potential value for their way of working. Second, cognitive participation was promising, as there was much interest coming from the PHCPs to support and start working with a GOC approach. Third, relating to collective action, PHCPs in the sample believed that GOC can easily be integrated into existing work without disrupting any working relationships with colleagues or patients. However, the resources needed to integrate GOC in their practices were rather unclear. Fourth, reflexive monitoring will be an area that needs consideration in a sustainable implementation plan, as participants scored it rather low. PHCPs should be encouraged to reflect and share experiences about how to use GOC in their daily practices. In short, the constructs collective action and reflexive monitoring presented mixed results, which suggests that these mechanisms should still be strengthened in implementation efforts to facilitate GOC in primary care.

The *general questions of the NoMAD survey* showed that more than half of the participants felt that GOC was already a normal part of their work. Looking at the literature, we learn that PHCPs often self-assess their practices as being in line with GOC, while they are in fact still focusing on health-related goals of their patients instead of life goals (Purkaple *et al*., 2020; Berntsen *et al*., 2015). For our study, we did not collect data on how the participants would apply GOC in their practice. Future work should assess whether GOC was implemented after these meetings, and whether, with time, the collective action and reflexive monitoring mechanisms are important to that implementation. In our view, it is not surprising that coherence and cognitive participation are more likely to be triggered in an earlier stage (even pre-implementation), while collective action and reflexive monitoring are triggered in a later stage of implementation. First, the sense-making and relation work is needed to grasp a full understanding of the concept before concrete actions and monitoring can take place. This was also found in similar NPT studies (Alharbi *et al*., 2014).

The construct of *coherence* showed that most of the participants saw the potential of GOC but reported that a shared understanding on the concept could be improved. Further growth in understanding could be facilitated through interprofessional collaboration. As in the construct of *cognitive participation*, participants were open to working with colleagues to implement GOC, this interprofessional collaboration could be a step forward toward increasing understanding (Bookey-Bassett *et al*., 2017). One of the benefits of interprofessional collaboration is the fact that PHCPs with more knowledge on GOC could be appointed as champions to initiate and disseminate GOC in their practice and thus enhance *cognitive participation.* From our expectations, greater understanding or adoption of GOC as a common philosophy within an interprofessional context could also be beneficial to strengthening relational integration, leading to more *collective action.* Other studies confirm that GOC could enhance collaboration in an interprofessional context and help PHCPs in their decision-making (Mold *et al*., 1991; Mangin *et al*., 2016). To further enhance *collective action*, policy and management activities must support PHCPs in implementing GOC in their work. As the participants did not perceive having this supportive context, it is important to make this one of the focal points for the future to make the implementation of GOC successful.

An interprofessional context could facilitate learning from each other as a means. To enable *reflexive monitoring*, it is key to initiate formal or informal moments where PHCPs can reflect upon GOC after they have been given the chance to integrate the approach in their practices (van Dongen *et al*., 2016). This could provide opportunities to share experiences about cases in which they intentionally applied GOC and how this was assessed. In this way, PHCPs could see the value of GOC from others and incorporate feedback from others into their practice.

We recognize the importance of interprofessional collaboration throughout the findings of all four constructs. NPT allowed us to identify the gaps to promote integration and embedding of GOC in primary care but did not propose guidance on how to overcome those gaps. Therefore, we suggest to develop interprofessional training so PHCPs can learn from and with each other and to build up a common ground on GOC which enhances collective action and reflexive monitoring. While there is overall recognition that interprofessional training is needed, there is no consensus in how this training should be organized and what content it should include. Disagreement exists about the population that should be targeted by the training, the duration of the training, and the methods that need to be used (Reuben and Jennings, 2019; Lusk and Fater, 2013). There is also disagreement about the exact skills that need to be taught, although the development of certain personal skills (e.g., communication skills for active listening) is probably one of the cornerstones (Kuluski *et al*., 2019; Reuben and Jennings, 2019; Vermunt et al., 2017; Poitras *et al*., 2018). It is also recommended that GOC be translated into a more concrete intervention with specific guidelines to enable deeper understanding of the concept along with tools to provide GOC.

### Strengths and limitations

This study captures how GOC is accepted and interpreted by PHCPs who have been introduced to the approach in a local interprofessional community meeting. A strength of this study is the diversity of the sample of professions, which captures the interprofessional nature of primary care settings in which GOC is introduced. However, no statistical comparison between professions was executed as the sample sizes were too small.

For this study, it is possible that participants might have shown more affinity or openness toward GOC as they voluntarily participated in this meeting. The study should be described as an exploratory study with PHCPs interested in GOC, which can contribute to determining and targeting further implementation strategies. However, as the survey was launched immediately after the community meeting, PHCPs did not have the opportunity to apply GOC in their daily practice. It was therefore too early to measure several of the NPT constructs, especially reflexive monitoring. However, it is remarkable that coherence and cognitive participation were already triggered after following one single interprofessional meeting on GOC. At the same time, it is not surprising that there is a lack of collective action and reflexive monitoring given such limitation of implementation effort. This is powerful given GOC represents a paradigm shift in how care is to be delivered and perhaps could be the most significant hurdle.

Future research should focus on the follow-up with the participants after these meetings to see what sticks and what has dropped to guide the research outlines.

## Conclusion

This study is a first step toward exploring the implementation gaps of GOC. It helps to define a strategy for implementing a GOC intervention and states the importance of developing interprofessional training. Initial results on how GOC is perceived by PHCPs are promising. Participants of the interprofessional community meeting recognized the value of GOC and showed willingness to apply the approach in their practices. This suggests that there is a foundation for further implementation efforts to successfully establish GOC as a normalized approach for patients with chronic conditions in primary care. To further enable GOC to become routine practice, several mechanisms of the NPT still require attention. Development of a concrete intervention will enable PHCPs to have a deeper understanding in how to get started with GOC.
